# Air Pollution from Road Traffic and Systemic Inflammation in Adults: A Cross-Sectional Analysis in the European ESCAPE Project

**DOI:** 10.1289/ehp.1408224

**Published:** 2015-03-27

**Authors:** Timo Lanki, Regina Hampel, Pekka Tiittanen, Silke Andrich, Rob Beelen, Bert Brunekreef, Julia Dratva, Ulf De Faire, Kateryna B. Fuks, Barbara Hoffmann, Medea Imboden, Pekka Jousilahti, Wolfgang Koenig, Amir A. Mahabadi, Nino Künzli, Nancy L. Pedersen, Johanna Penell, Göran Pershagen, Nicole M. Probst-Hensch, Emmanuel Schaffner, Christian Schindler, Dorothea Sugiri, Wim J.R. Swart, Ming-Yi Tsai, Anu W. Turunen, Gudrun Weinmayr, Kathrin Wolf, Tarja Yli-Tuomi, Annette Peters

**Affiliations:** 1 Department of Health Protection, National Institute for Health and Welfare, Kuopio, Finland; 2 Institute of Epidemiology II, German Research Center for Environmental Health, Helmholtz Zentrum München, Neuherberg, Germany; 3 Biometry and Epidemiology, Institute for Medical Informatics, University Hospital Essen, University of Duisburg-Essen, Essen, Germany; 4 Institute for Risk Assessment Sciences, Utrecht University, Utrecht, the Netherlands; 5 Julius Center for Health Sciences and Primary Care, Department of Epidemiology, University Medical Center Utrecht, Utrecht, the Netherlands; 6 Swiss Tropical and Public Health Institute, Basel, Switzerland; 7 University of Basel, Basel, Switzerland; 8 Institute of Environmental Medicine, Karolinska Institutet, Stockholm, Sweden; 9 IUF-Leibniz Research Institute for Environmental Medicine, Düsseldorf, Germany; 10 Medical Faculty, Deanery of Medicine, Heinrich Heine University Düsseldorf, Düsseldorf, Germany; 11 Department of Chronic Disease Prevention, National Institute for Health and Welfare, Helsinki, Finland; 12 Department of Internal Medicine II-Cardiology, University of Ulm Medical Center, Ulm, Germany; 13 Deutsches Herzzentrum München, Technische Universität München, Munich, Germany; 14 West-German Heart Center, Department of Cardiology, University of Duisburg-Essen, Essen, Germany; 15 Department of Medical Epidemiology and Biostatistics, Karolinska Institutet, Stockholm, Sweden; 16 Centre for Environmental Health, National Institute for Public Health and the Environment, Bilthoven, the Netherlands; 17 Department of Environmental and Occupational Health Sciences, University of Washington, Seattle, USA; 18 Institute of Epidemiology and Medical Biometry, Ulm University, Ulm, Germany

## Abstract

**Background:**

Exposure to particulate matter air pollution (PM) has been associated with cardiovascular diseases.

**Objectives:**

In this study we evaluated whether annual exposure to ambient air pollution is associated with systemic inflammation, which is hypothesized to be an intermediate step to cardiovascular disease.

**Methods:**

Six cohorts of adults from Central and Northern Europe were used in this cross-sectional study as part of the larger ESCAPE project (European Study of Cohorts for Air Pollution Effects). Data on levels of blood markers for systemic inflammation—high-sensitivity C-reactive protein (CRP) and fibrinogen—were available for 22,561 and 17,428 persons, respectively. Land use regression models were used to estimate cohort participants’ long-term exposure to various size fractions of PM, soot, and nitrogen oxides (NO_x_). In addition, traffic intensity on the closest street and traffic load within 100 m from home were used as indicators of traffic air pollution exposure.

**Results:**

Particulate air pollution was not associated with systemic inflammation. However, cohort participants living on a busy (> 10,000 vehicles/day) road had elevated CRP values (10.2%; 95% CI: 2.4, 18.8%, compared with persons living on a quiet residential street with < 1,000 vehicles/day). Annual NO_x_ concentration was also positively associated with levels of CRP (3.2%; 95% CI: 0.3, 6.1 per 20 μg/m^3^), but the effect estimate was more sensitive to model adjustments. For fibrinogen, no consistent associations were observed.

**Conclusions:**

Living close to busy traffic was associated with increased CRP concentrations, a known risk factor for cardiovascular diseases. However, it remains unclear which specific air pollutants are responsible for the association.

**Citation:**

Lanki T, Hampel R, Tiittanen P, Andrich S, Beelen R, Brunekreef B, Dratva J, De Faire U, Fuks KB, Hoffmann B, Imboden M, Jousilahti P, Koenig W, Mahabadi AA, Künzli N, Pedersen NL, Penell J, Pershagen G, Probst-Hensch NM, Schaffner E, Schindler C, Sugiri D, Swart WJ, Tsai MY, Turunen AW, Weinmayr G, Wolf K, Yli-Tuomi T, Peters A. 2015. Air pollution from road traffic and systemic inflammation in adults: a cross-sectional analysis in the European ESCAPE project. Environ Health Perspect 123:785–791; http://dx.doi.org/10.1289/ehp.1408224

## Introduction

Exposure to ambient particulate matter pollution (PM) affects both respiratory and cardiovascular health, and is a major contributor to the global burden of disease (Lim et al. 2012). One of the main sources of particulate air pollution is road traffic. Tailpipe exhaust is a source of fine combustion particles (soot), which are associated with cardiorespiratory health effects and premature mortality (Janssen et al. 2012). However, road traffic also produces road dust consisting mainly of coarse particles. There is increasing evidence on the harmfulness of coarse particulate matter (Brunekreef and Forsberg 2005).

Ischemic heart disease is the leading contributor to the burden of disease worldwide, and stroke is ranked third (Murray et al. 2012). An American Heart Association Statement has concluded that exposure to fine particulate air pollution is likely to be causally linked to cardiovascular disease (CVD) (Brook et al. 2010). Because of its ubiquitous presence, particulate air pollution is a relevant trigger of myocardial infarction on a population level (Nawrot et al. 2011). There is less, but increasing, evidence on the associations between long-term exposure to traffic-related air pollutants and the incidence of CVD (Brook et al. 2010).

It has been hypothesized that increased systemic inflammation—a risk factor for, as well as a consequence of, atherosclerosis—is the main underlying mechanism linking long-term PM exposure to incidence of CVD (Brook et al. 2010). A number of studies have associated daily variation in PM with short-term changes in the concentrations of circulating inflammatory markers (Brook et al. 2010). However, it is not known whether recurrent high short-term exposures may also lead to chronic and partly self-sustained systemic inflammation. Associations between long-term within-city variation in PM, typically driven by traffic emissions, and systemic inflammation have been evaluated only in a few population-level studies, and with conflicting results (Chuang et al. 2011; Forbes et al. 2009; Hoffmann et al. 2009; Panasevich et al. 2009; Schwartz 2001). Therefore, we evaluated within the large multi-center ESCAPE project (European Study of Cohorts for Air Pollution Effects) the associations of long-term exposure to traffic and traffic-related air pollution with high-sensitivity C-reactive protein (CRP), a clinically established sensitive marker of systemic inflammation (Emerging Risk Factors Collaboration et al. 2010), and fibrinogen, a hemostatic factor with proinflammatory properties.

## Methods

*Study population*. The ESCAPE project linked data from existing cohorts in different parts of Europe with modeled long-term residential air pollution concentrations. The studies were approved by the local ethical committees and conducted according to the guidelines laid down in the Declaration of Helsinki. All data were collected in accordance with the Declaration of Helsinki, and all participants gave an informed consent before the study. Data on blood levels of high-sensitivity CRP was available for five cohorts in Northern and Central Europe: the National FINRISK study in Finland, KORA (Cooperative Health Research in the Region of Augsburg) and HNR (Heinz Nixdorf Recall) in Germany, SAPALDIA (Swiss Study on Air Pollution and Lung and Heart Disease In Adults) in Switzerland, and TwinGene in Sweden. For the analyses of fibrinogen, four cohorts were available: FINRISK, KORA, HNR, and the “60-year-olds” study in Sweden. Descriptive statistics of these cohorts are presented in Table 1. See Supplemental Material, “Cohort descriptions” and “Biochemical measurements,” for further information on the cohorts, as well as the methods for estimating CRP and fibrinogen.

**Table 1:** Descriptive statistics for the study cohorts.

Study cohort (country)	Time of baseline visit	Age^*a*^		Sex^*a*^	BMI^*a*^	Smoking^*a*^	C-reactive protein		Fibrinogen	
		Range (years)	Average (years)	Female (%)	Average (kg/m^2^)	Current (%)	*n* of samples	Average ± SD (mg/L)	*n* of samples	Average ± SD (g/L)
KORA (Germany)	1994–1995, 2000–2001	25–74	50.2	50.1	27.2	23.9	7,137	2.7 ± 4.9	7,151	2.76 ± 0.67
HNR (Germany)	2000–2003	45–76	59.5	49.9	27.9	23.3	4,492	3.1 ± 9.0	4,444	3.3 ± 0.8
SAPALDIA (Switzerland)	2002	18–60	53.8	52.7	25.5	27.7	1,688	2.1 ± 3.2	NA	NA
FINRISK (Finland)	1997, 2002, 2007	25–64	48.9	52.4	26.6	28.3	7,627	2.3 ± 4.5	2,044	3.55 ± 0.81
TwinGene (Sweden)	2004–2008	47–90	64.2	56.2	25.2	19.1	1,617	2.8 ± 4.9	NA	NA
60-year-olds (Sweden)	1997–1999	60	60.4	52	26.8	20.8	NA	NA	3,789	3.02 ± 0.77
Abbreviations: BMI, body mass index; NA, not available. ^***a***^Numbers are based on participants with CRP measurements (except for 60-year-olds cohort). Descriptive statistics were similar when limited to participants with fibrinogen data (data not shown).										

*Exposure data*. Air pollution measurements and modeling followed common study protocols (ESCAPE 2008) and were coordinated by the Institute for Risk Assessment Sciences of Utrecht University. Concentrations of several size fractions of particulate air pollution (PM_2.5_, aerodynamic diameter < 2.5 μm; PM_10_, < 10 μm; PM_coarse_, 2.5 μm ≤ diameter < 10 μm), black carbon (PM_2.5_ absorbance), that is, soot, and nitrogen oxides (NO_2_ and NO_x_) were measured (Cyrys et al. 2012; Eeftens et al. 2012b). In each study area, pollutants were measured in different locations for 2 weeks during winter, summer, and an intermediate season; that is, 6 weeks altogether in an approximately 1-year period (starting from autumn 2008 for KORA, HNR, and SAPALDIA; from winter 2008 for TwinGene and 60-year-olds study; and from winter 2010 for FINRISK). At the same time, pollutants were measured continuously for 1 year at one regional or urban background site in each study area as described by Eeftens et al. (2012b). Based on these measurements, annual outdoor concentrations of air pollution were modeled at the participants’ home addresses using land use regression (LUR) models (Beelen et al. 2013; Eeftens et al. 2012a). The models were developed for each cohort based on a common manual (ESCAPE 2008). A large number of potential predictors of air pollution concentrations derived from geographic information systems (e.g., traffic intensity, population density, forms of land use) were tested in the models with an aim to maximize explained variability. In SAPALDIA, models for PM were only available for one study area (Lugano, Switzerland). Measurements of air pollution for modeling purposes were conducted later (2008–2011) than the collection of blood samples (1994–2008). Therefore, historical air pollution concentrations were extrapolated for sensitivity analyses using data from routine monitoring network site(s) as explained by Beelen et al. (2014).

In addition to modeled air pollution concentration, two indices of exposure to traffic were used: traffic intensity (vehicles/day) at the road nearest to the participant’s residence, and traffic load on major roads within 100 m of the residence (measured as the product of traffic intensity and the length of major road fragments within a 100-m buffer). Databases on traffic intensity were constructed for each cohort based on measurements conducted by county- or municipal-level authorities (Eeftens et al. 2012a). The traffic indices were always included in the statistical models together with background NO_2_ concentrations. Information on the traffic intensity at the nearest road was not available for the HNR cohort.

*Data analyses*. One blood sample per person was available for the analyses, taken at the baseline visit except for SAPALDIA (first follow-up visit). High-sensitivity CRP and fibrinogen on continuous scales were used for the current cross-sectional analyses. Based on the distribution of inflammatory blood marker concentrations and model residuals, the (natural) logarithmic value of CRP concentrations was used in the analyses, whereas fibrinogen concentrations were not transformed. Linear regression models in SAS (SAS Institute Inc.) and STATA (StataCorp) software were used for the analyses. In the meta-analyses, cohort-specific effects were treated as having a random component apart from sampling error, and pooled using the empirical Bayes method. Heterogeneity of the cohort-specific estimates was estimated using Cochrane’s Q test. Effect estimates are presented as percent difference from the outcome mean together with 95% confidence interval (CI), and are calculated for an increase of 5 μg/m^3^ in PM_2.5_ and PM_coarse_, 10 μg/m^3^ in PM_10_ and NO_2_, 20 μg/m^3^ in NO_x_, and 1 × 10^–5^/m in PM_2.5_ absorbance. An association was deemed present when it was statistically significant (*p* < 0.05).

Confounders in the statistical models were selected *a priori* based on literature on the determinants of CRP and fibrinogen concentrations. Four different regression models were used to calculate the effect estimates. All these models included, in addition to exposure variables, an indicator variable for baseline visit for FINRISK and KORA, which had more than one recruitment period, and an area indicator for FINRISK, which consisted of two clearly separate study areas. Additional variables controlled for in the first regression model (the crude model) were age and sex. The second model (the main model) also included education (primary school or less, up to secondary school or equivalent, university degree), body mass index, smoking status (current smoker, ex-smoker, never smoker), physical activity (≤ 3 times/month, once a week, 2–3 times/week, > 3 times/week), and alcohol intake (number of drinks per week; 0, 1–3, 4–6, > 6). Effect estimates were also calculated using an extended model, which included additional covariates not available in all cohorts (alcohol intake excluding wine, wine intake, household income, fish intake, fruit intake, meat intake, CVD, diabetes, arthritis, ulcerative colitis, passive smoking).

The fourth model was a mixed model that included the same covariates as the main model, but also included neighborhood level as a random effect to take into account possible spatial clustering of observations. Depending on data availability for each cohort, neighborhoods either were defined as grids or were based on ZIP code areas. We assumed that the effect of neighborhood on health was mainly attributable to area level socioeconomic status. Therefore, area level median household income or unemployment rate were included in the model as fixed effects.

Effect modification by obesity (cutpoint, 30 kg/m^2^ for body mass index) and sex was evaluated by adding an interaction term in the main model. In addition, a number of sensitivity analyses were conducted using the main model: exclusion of participants with high (> 10 mg/L) CRP values that may indicate a current bacterial infection, exclusion of study participants who moved residence during the 2 years before blood draw (information not available for KORA and for HNR participants from Bochum, Germany; see Supplemental Material, “Cohort descriptions”), use of back-extrapolated concentrations of NO_x_, NO_2_, and PM_10_ (in cohorts where changes over time in concentrations had occurred), inclusion of indicators for medication use (statins and antihypertensives), and finally inclusion of traffic noise for a subset of data in each center [data available only for larger population agglomerates as defined by the European Union (EU), no data available for SAPALDIA]. Traffic noise was modeled according to the guidelines of the EU noise directive (EU 2002). When using meta-analyses to generate combined effect estimates for sensitivity analyses, we substituted effect estimates from the main model for cohorts missing data for the sensitivity analyses.

## Results

In the participating cohorts, the average age of the participants ranged from 49 to 64 years, and the proportion of current smokers ranged from 19 to 28% (Table 1). All cohorts consisted of approximately 50% women. There were altogether 22,561 participants with measures of CRP, and 17,428 samples of fibrinogen. Consistently across the cohorts, the standard deviation was much larger for CRP than for fibrinogen.

Modeled air pollution concentrations were the lowest in the Northern cohorts, in Finland and Sweden (Table 2). The highest traffic loads were observed near (within 100 m) the homes of the HNR and SAPALDIA study participants, and the lowest in the KORA cohort.

**Table 2:** Descriptive statistics for modeled annual residential air pollution concentrations and indices of traffic intensity.

Study cohort	PM_2.5_ (μg/m^3^)			PM_10_ (μg/m^3^)			PM_coarse_ (μg/m^3^)			PM_2.5_ absorbance (1 × 10^–5^/m)			NO_2_ (μg/m^3^)			NO_x_ (μg/m^3^)			Traffic, nearest road^*a*^ (vehicles/day)		Traffic, within 100 m^*b*^ (vehicles/day × m)	
	Avg	5th Pctl	95th Pctl	Avg	5th Pctl	95th Pctl	Avg	5th Pctl	95th Pctl	Avg	5th Pctl	95th Pctl	Avg	5th Pctl	95th Pctl	Avg	5th Pctl	95th Pctl	Avg	95th Pctl	Avg	95th Pctl
KORA	13.6	12.5	15.3	20.3	16.5	24.3	6.2	4.9	8.4	1.7	1.5	2	18.7	13.7	25.9	32.6	23.8	47.1	1,600	8,500	434,000	2,790,000
HNR	18.4	16.9	20.4	27.8	25.3	31.6	10	7.3	12.4	1.6	1.2	2.2	30.3	23.4	38.7	50.9	33.6	71.9	NA	NA	1,124,000	4,432,000
SAPALDIA	17.1	14.7	19.9	23.7	19.8	27.4	6.7	4.9	8.6	2	1.3	2.5	27.8	17.1	37.9	46.7	24.0	69.5	3,520	15,500	945,000	4,170,000
FINRISK	7.7	5.6	9.1	14.1	9.5	19.9	6.7	3.9	11	1.3	1.1	1.6	15.4	8.7	24.2	24.4	13.6	41.2	1,700	9,600	638,000	3,750,000
TwinGene	7.2	4.8	9.2	14.8	6.5	20.5	7.2	1.0	11.8	0.6	0.4	0.9	10.8	6.7	19.9	18.7	11.4	39.4	1,500	6,400	570,000	3,300,000
60-year-olds	7.3	4.9	9.2	15.0	6.8	20.6	7.3	1.3	11.8	0.6	0.4	1	10.7	6.5	20.3	18.6	12.0	39.3	1,400	6,300	519,000	3,090,000
Abbreviations: Avg, average; NA, not available; Pctl, percentile. ^***a***^Traffic intensity on the road nearest to the residence. ^***b***^Traffic load on major roads within 100 m from the residence.																						

**Figure 1:**
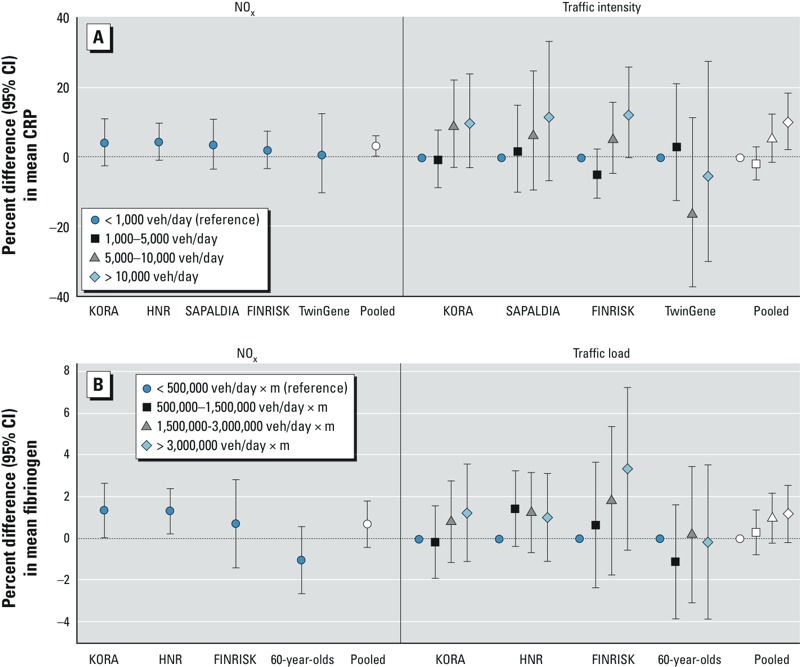
Cohort-specific and pooled effect estimates (the main model) for the associations of NO_x_ and traffic with inflammatory markers. veh, vehicles. Estimates calculated for a 20-μg/m^3^ increase in NO_x_. (*A*) NO_x_, traffic intensity at the nearest road, and CRP; (*B*) NO_x_, traffic load on major roads within 100 m, and fibrinogen.

In the pooled analyses using the main model, CRP serum levels were positively associated with annual NO_x_ concentration (3.2%; 95% CI: 0.3, 6.1 per 20-μg/m^3^ increase in NO_x_) and traffic intensity at the nearest road (10.2%; 95% CI: 2.4, 18.6 for the highest category, i.e., > 10,000 vehicles/day; reference category < 1,000 vehicles/day) without evidence of significant heterogeneity between ESCAPE cohorts (Table 3, Figure 1). Categories of traffic load within 100 m from residence were not consistently associated with CRP. Inclusion of an extended set of individual level confounders (Table 3, Extended model) had only minor effects on the estimates. When area-level confounding was taken into account, the effect estimate for NO_x_ decreased and was no longer statistically significant (2.5%; 95% CI: –0.6, 5.6) (Table 3, Area-level model). In contrast, effect estimates for traffic intensity at the nearest road were not affected. Particulate air pollution, including black carbon, and NO_2_ were not associated with CRP in the pooled analyses. However, in cohort-specific analyses, high effect estimates were observed for PM_2.5_ in the German cohorts: statistically nonsignificant for KORA (12.9%; 95% CI: –1.4, 29.2%; per 5-μg/m^3^ increase in PM_2.5_), but significant for HNR (16.1%; 95% CI: 0.5, 34.1%) (see Supplemental Material, Figure S1).

**Table 3:** Associations of air pollution and indices of traffic intensity with CRP and fibrinogen in the pooled analyses.

Exposures	Crude model^*a*^		Main model^*b*^		Extended model^*c*^		Area-level model^*d*^	
	% Diff^*e*^ (95% CI)	*p*_Heter_^*f*^	% Diff^*e*^ (95% CI)	*p*_Heter_^*f*^	% Diff^*e*^ (95% CI)	*p*_Heter_^*f*^	% Diff^*e*^ (95% CI)	*p*_Heter_^*f*^
CRP
PM_2.5_	9.4 (–9.7, 32.5)	0.00	2.4 (–7.5, 13.4)	0.05	2.3 (–6.4, 11.7)	0.11	–0.3 (–9.0, 9.2)	0.14
PM_10_	2.2 (–7.6, 12.9)	0.05	1.2 (–3.8, 6.4)	0.90	0.7 (–4.3, 5.9)	0.94	0.2 (–4.9, 5.6)	0.76
PM_coarse_	2.8 (–2.3, 8.2)	0.21	3.0 (–0.7, 6.8)	0.53	2.4 (–1.2, 6.1)	0.66	2.4 (–1.6, 6.5)	0.35
PM_2.5_ absorbance	3.2 (–6.9, 14.4)	0.01	0.4 (–5.2, 6.4)	0.73	0.0 (–5.5, 5.9)	0.86	–2.5 (–8.4, 3.6)	0.78
NO_2_	2.2 (–4.3, 9.2)	0.00	2.1 (–0.8, 5.1)	0.72	2.0 (–0.9, 4.9)	0.82	1.4 (–1.7, 4.6)	0.70
NO_x_	3.2 (–2.5, 9.2)	0.00	3.2 (0.3, 6.1)**	0.95	2.9 (0.1, 5.9)**	0.99	2.5 (–0.6, 5.6)	0.94
Traffic intensity at the nearest road (vehicles/day)
< 1,000	Reference		Reference		Reference		Reference
1,000–5,000	–0.8 (–5.9, 4.5)	0.81	–1.7 (–6.3, 3.2)	0.69	–2.0 (–6.6, 2.8)	0.81	–1.6 (–6.2, 3.3)	0.66
5,000–10,000	6.3 (–0.9, 14.1)*	0.57	5.5 (–1.2, 12.6)	0.42	4.5 (–2.0, 11.6)	0.55	5.1 (–2.7, 13.6)	0.35
> 10,000	12.8 (4.2, 22.2)**	0.84	10.2 (2.4, 18.6)**	0.77	9.8 (2.1, 18.2)**	0.84	10.2 (2.4, 18.6)**	0.76
Traffic load within 100 m on major roads (vehicles/day × m)
< 500,000	Reference		Reference		Reference		Reference
500,000–1,500,000	0.2 (–7.9, 9.0)	0.15	1.4 (–3.0, 6.0)	0.41	1.1 (–3.3, 5.7)	0.59	1.3 (–3.3, 6.1)	0.39
1,500,000–3,000,000	3.6 (–1.6, 9.1)	0.66	1.8 (–3.0, 6.8)	0.51	1.6 (–3.1, 6.5)	0.72	1.5 (–3.3, 6.5)	0.41
> 3,000,000	5.3 (–1.8, 13.0)	0.23	2.6 (–3.4, 9.0)	0.29	2.0 (–3.9, 8.3)	0.30	2.1 (–3.7, 8.3)	0.32
Fibrinogen
PM_2.5_	2.8 (0.3, 5.3)**	0.09	0.5 (–1.1, 2.0)	0.66	0.2 (–1.3, 1.8)	0.77	0.4 (–1.3, 2.0)	0.96
PM_10_	0.5 (–2.3, 3.2)	0.01	0.1 (–1.4, 1.8)	0.18	0.0 (–1.4, 1.4)	0.27	–0.2 (–1.4, 1.0)	0.39
PM_coarse_	–0.0 (–1.7, 1.6)	0.03	–0.1 (–1.2, 0.9)	0.24	–0.3 (–1.2, 0.6)	0.37	–0.5 (–1.4, 0.4)	0.80
PM_2.5_ absorbance	1.5 (–0.4, 3.5)	0.25	0.3 (–1.2, 1.9)	0.36	0.1 (–1.3, 1.6)	0.49	–0.5 (–2.0, 1.1)	0.61
NO_2_	0.8 (–1.2, 2.8)	0.00	0.5 (–0.8, 1.8)	0.04	0.5 (–0.7, 1.6)	0.10	0.4 (–0.6, 1.3)	0.30
NO_x_	0.9 (–0.9, 2.7)	0.00	0.7 (–0.4, 1.8)	0.08	0.6 (–0.5, 1.6)	0.13	0.5 (–0.4, 1.3)	0.31
Traffic intensity at the nearest road (vehicles/day)
< 1,000	Reference		Reference		Reference		Reference
1,000–5,000	0.5 (–0.7, 1.8)	0.43	0.2 (–1.5, 1.8)	0.28	0.0 (–1.2, 1.3)	0.48	0.1 (–1.4, 1.7)	0.29
5,000–10,000	1.8 (0.0, 3.6)*	0.93	1.8 (0.1, 3.5)**	0.99	1.6 (–0.1, 3.3)*	0.93	1.9 (0.1, 3.6)**	0.99
> 10,000	1.3 (–0.7, 3.3)	0.40	0.9 (–1.0, 2.8)	0.84	0.6 (–1.3, 2.5)	0.75	0.9 (–1.1, 2.8)	0.85
Traffic load within 100 m on major roads (vehicles/day × m)
< 500,000	Reference		Reference		Reference		Reference
500,000–1,500,000	0.2 (–1.3, 1.7)	0.18	0.3 (–0.8, 1.4)	0.39	0.1 (–0.9, 1.2)	0.37	0.2 (–1.0, 1.4)	0.32
1,500,000–3,000,000	1.4 (0.2, 2.7)**	0.87	1.0 (–0.2, 2.2)*	0.87	0.9 (–0.3, 2.1)	0.86	1.0 (–0.2, 2.2)	0.86
> 3,000,000	1.9 (0.3, 3.5)**	0.35	1.2 (–0.2, 2.6)*	0.59	1.1 (–0.2, 2.5)	0.64	1.2 (–0.2, 2.6)*	0.57
Effect estimates were calculated for a change of 5 μg/m^3^ in PM_2.5_ and PM_coarse_, 10 μg/m^3^ in PM_10_ and NO_2_, 20 μg/m^3^ in NO_x_, and 1 x 10^–5^/m in PM_2.5_ absorbance. ^***a***^Model was adjusted for age, sex, baseline visit (KORA and FINRISK), study area (FINRISK). ^***b***^Model was adjusted for age, sex, baseline visit (KORA and FINRISK), study area (FINRISK), education, body mass index, smoking status, physical activity, alcohol intake. ^***c***^Model was adjusted for age, sex, baseline visit (KORA and FINRISK), study area (FINRISK), education, body mass index, smoking status, physical activity in all cohorts, and for wine intake, intake of other types of alcohol, household income, fish intake, fruit intake, meat intake, CVD, diabetes, arthritis, colitis ulcerosa, passive smoking in cohorts where the data available. ^***d***^Mixed model (neighborhood as a random effect) was adjusted for age, sex, baseline visit (KORA and FINRISK), study area (FINRISK), education, body mass index, smoking status, physical activity, and area level household income or unemployment rate. ^***e***^Percent difference. ^***f***^*p*-Value for heterogeneity. *Effect estimates with *p*-values < 0.1.**Effect estimates with *p*-values < 0.05.								

In the pooled analyses for fibrinogen, borderline significant (*p* < 0.1) positive associations were observed for NO_x_ and traffic load within 100 m (in the two highest categories) without evidence of heterogeneity (Table 3, Figure 1). Slightly decreased effect estimates for traffic load were observed when the extended model was used (Table 3). Adding area-level confounders in the models did not affect the results. Other air pollutants were not associated with fibrinogen. However, in cohort-specific analyses, high effect estimates were observed for NO_2_ in the German cohorts: statistically significant for KORA (1.6%; 95% CI: 0.5, 2.7% per 10-μg/m^3^ increase in NO_2_), but nonsignificant for HNR (1.2%; 95% CI: –0.2, 2.7%) (see Supplemental Material, Figure S2). Finally, the middle category of traffic intensity was positively associated with fibrinogen in the pooled analyses, but there was no evidence of association for the other categories.

Estimated effects of air pollution or traffic were not modified by obesity or sex: The confidence intervals were overlapping in the pooled analyses, and no consistent patterns were observed in the cohort-specific analyses (data not shown). Inclusion of environmental noise in the main model for CRP increased effect estimates for traffic intensity in the two highest categories (8.2%; 95% CI: 1.0, 15.9, and 11.9%; 95% CI: 3.4, 21.2, respectively) (see Supplemental Material, Table S1). Inclusion of information on statin use in the model and exclusion of persons who had recently moved led to decreased estimated effects of NO_x_ on CRP (2.5%; 95% CI: –0.4, 5.4, and 2.3%; 95% CI: –1.0, 5.8, respectively).

After exclusion of unusually high CRP values (237 observations for KORA, 181 for HNR, 47 for SAPALDIA, 257 for FINRISK, and 62 for TwinGene), effect estimates increased for PM_coarse_ (from 3.0%; 95% CI: –0.7, 6.8 to 6.4%; 95% CI: –0.4, 13.5% for a 5-μg/m^3^ increase).There were only minor changes in the effect estimates, when back-extrapolated air pollution levels were used in the models for CRP and fibrinogen (data not shown). In general, sensitivity analyses had only minor effects on the effect estimates for fibrinogen (see Supplemental Material, Table S1).

## Discussion

In this European multi-center study, modeled annual particulate air pollution concentrations were not associated with systemic inflammation. However, high traffic intensity on the road nearest to home was associated with increased levels of high-sensitivity CRP, an established marker for systemic inflammation. In the main analyses, long-term exposure to NO_x_, an indicator for vehicle exhaust, was also associated with CRP serum levels. However, the effect estimate was more sensitive to further adjustments in the model, such as inclusion of area-level confounders. Fibrinogen was not consistently associated with air pollution, and there was only limited support for a positive association with traffic exposure.

CRP is an acute-phase reactant that has been shown in many cohort studies to be a reliable measure of underlying systemic inflammation and a predictor of future myocardial infarction and stroke (Danesh et al. 2004; Emerging Risk Factors Collaboration et al. 2010). In the present study, NO_x_ was the only pollutant that was positively associated with CRP in the analyses performed with the *a priori*–selected main model. However, the association between NO_x_ and CRP was somewhat sensitive to adjustments in the models, such as inclusion of area-level confounders or information on statin use or moving history. Therefore, further studies are needed to confirm the suggestive association. Furthermore, it is possible that nitrogen oxides are not causally related to systemic inflammation, but that they rather represent markers for traffic-related air pollution mixture, including ultrafine particles, volatile organic compounds, and traffic-related PM_2.5_.

In the present study, no evidence of an effect was found for soot, measured as PM_2.5_ absorbance, which is considered to be a key component of the near-road traffic-related pollution mixture. One reason for this might be that soot is stable in the atmosphere and is therefore associated not only with fresh but also with aged vehicle exhaust, which may be less toxic due to secondary atmospheric reactions. On the other hand, NO_x_ is a better marker for vehicle exhausts than soot, which also has other (combustion) sources such as residential wood combustion (Healy et al. 2012; Janssen et al. 2012).

Another traffic-related pollutant is the coarse size fraction of particulate matter, which forms the major part of road dust. Road dust is rich in earth minerals (wear of road surface), transition metals (wear of brakes and clutch), and biological material (pollen fragments, bacteria). All these components have been shown in toxicological studies to have considerable proinflammatory potential (Camatini et al. 2012; Jalava et al. 2009; Schwarze et al. 2007). In the present study, effect estimates for coarse particles were consistently positive, but statistical significance was not reached in the main analyses. This could be attributable partly to exposure misclassification: LUR models predicted less of the variance of outdoor concentrations of PM_coarse_ (average *R*^2^ of models = 71%) than of NO_x_ (*R*^2^ = 86%) (Beelen et al. 2013; Eeftens et al. 2012a).

In the pooled analyses, markers of inflammation were not associated with PM_2.5_ or PM_10_, the spatial variation of which is less strongly associated with road traffic than that of NO_x_ (Eeftens et al. 2012b). It can be speculated that PM_2.5_ and PM_10_ contain some components that are less toxic than pollution mixture near traffic. Only a few epidemiological studies have previously evaluated associations between long-term exposure to air pollution and CRP. In a cross-sectional study conducted in a representative sample of the English population (Forbes et al. 2009), no association was observed between CRP and PM_10_ or NO_2_. Fibrinogen was not associated with air pollution either. In the study, dispersion models were used to estimate air pollution concentrations for the centroid (1 km^2^) of each postcode sector; the same exposure estimate was assigned to all study participants within the sector. The spatial resolution of exposure assessment was higher in the present study, which might explain the discrepancy concerning CRP. In a German study that used the HNR cohort and dispersion models, PM_2.5_ was found to be associated with CRP in men but not in women (Hoffmann et al. 2009). In the present study, PM_2.5_ was associated with CRP in the HNR cohort in men and women combined. In an earlier cross-sectional study conducted in Stockholm, Sweden (Panasevich et al. 2009), there was some evidence for an association between dispersion-modeled long-term NO_2_ concentration and CRP, but not for fibrinogen, among adults (45–70 years of age). In the same study, NO_2_ was also associated with another marker of systemic inflammation, interleukin-6. In a cross-sectional study conducted in a representative sample of adults ≥ 54 years of age in Taiwan (Chuang et al. 2011), long-term PM_10_, PM_2.5_, and NO_2_ concentrations were all associated with interleukin-6.

Fibrinogen is a hemostatic factor with proinflammatory properties. It has been shown that inflammatory and coagulatory processes are intertwined via many molecular mechanisms, and typically systemic inflammation is associated with increased levels of fibrinogen (Davalos and Akassoglou 2012). One previous cross-sectional study, conducted in a representative sample of the U.S. population (Schwartz 2001), had data on fibrinogen but not on CRP. Both NO_2_ and PM_10_ were found to be associated with fibrinogen as well as with platelet counts. In the present study, fibrinogen was not associated with the studied pollutants. Compared with CRP, there were fewer observations (17,428 vs. 22,561) and lower variability in concentrations—both factors that reduce the chance of identifying associations.

The most robust association in the present study was observed between traffic intensity at the road nearest to home and CRP. Cohort participants living on a busy road (> 10,000 vehicles/day) had significantly increased CRP compared with participants living on a quiet residential street (< 1,000 vehicles/day). The estimated increase was modest (10.2%), but can be seen as clinically significant considering that CVD risk seems to increase linearly with increasing CRP (Ridker and Cook 2004). It is noteworthy that increased CRP concentration is not necessarily a causal factor in the development of CVD, but might only be an indicator of an increased risk. No statistically significant association was observed between CRP levels and traffic load within 100 m on major roads, another indicator of proximity to dense traffic, perhaps because traffic intensity at the nearest road indicates better exposure to fresh traffic exhaust. An earlier cross-sectional study conducted among adult Puerto Ricans (45–75 years of age) has reported an association between living within 100 m from busy roads (> 20,000 vehicles/day) and CRP (Rioux et al. 2010). In the present study, positive but statistically nonsignificant effect estimates were observed for high traffic load.

Inhalation of particulate air pollution is known to cause pulmonary oxidative stress and pulmonary inflammation (Brook et al. 2010), which in turn has been hypothesized to lead further to systemic inflammation. It has been recently shown that oxidative potential of particles sampled near major streets is several times higher than of particles at urban background locations (Boogaard et al. 2012). Systemic inflammation and impaired endothelial function provide a plausible link between exposure to traffic-related pollutants and CVD. Systemic inflammation plays a major role in the development of atherosclerosis, possibly leading, over time, to clinically detectable CVD (Libby et al. 2002). Acute increases in the inflammatory response and the development of vulnerable arterial plaque may in turn lead to acute exacerbations of CVD. An increasing number of studies link either traffic-related pollutants or traffic intensity with the prevalence, and even incidence, of CVD (Brook et al. 2010).

Most of the evidence on the health effects of PM relates to cardiorespiratory health. However, some recent studies have associated long-term PM exposure with additional outcomes such as rheumatoid arthritis, diabetes, cognitive impairment, and leukemia (Amigou et al. 2011; Hart et al. 2009; Krämer et al. 2010; Ranft et al. 2009). Importantly, an inflammatory component is part of the pathogenesis of many major noncommunicable diseases such as diabetes, Alzheimer’s disease, and cancer (Mantovani et al. 2008; Sardi et al. 2011). This adds to the importance of evaluating links between exposure to air pollution from local traffic and systemic inflammation.

As part of the large European ESCAPE project, this study has some major strengths, including the large number of observations, a multi-city study design covering many types of exposure environments and climates, rigorous exposure assessment including extensive quality control measures, and the use of common study manuals for exposure assessment, data management, and data analyses. An obvious limitation is the cross-sectional nature of the study. However, a large number of individual-level and some neighborhood-level covariates were available for inclusion in the statistical models to reduce confounding. It was unfortunate that data on individual-level income, a quantitative indicator of socioeconomic status, was not available. However, we believe that residual confounding by individual level socioeconomic status would have been substantially reduced by adjusting for educational level and lifestyle factors including smoking. The association between traffic intensity at the road nearest to home and CRP was not sensitive to model specifications, which increases the plausibility of our main finding.

Other limitations include the retrospective nature of exposure assessment, varying LUR model performance for different air pollution components, and lack of data on personal exposures. Several recent studies have demonstrated that spatial variation in air pollution exposure can be reliably estimated also retrospectively, even when overall mean levels may change over time (Eeftens et al. 2011; Gulliver et al. 2013). However, the differences in the accuracy of exposure assessment among pollutants should be taken into account when interpreting the present results. Therefore, even though no statistically significant associations were observed either for PM_2.5_ or coarse particles, the apparent lack of association for the latter could be attributable partly to the difficulty of predicting concentrations. Finally, there is evidence that living close to traffic contributes significantly to personal exposures (Van Roosbroeck et al. 2008). Therefore, indices of traffic intensity near home can be considered as reasonable proxies for exposure to traffic-related air pollution. This is especially true for traffic intensity on the road nearest to home, because emissions occur close to exposed persons, so more complex physicochemical dispersion processes do not affect the concentrations.

## Conclusions

Particulate air pollution was not associated with two markers of systemic inflammation in the present analysis. However, compared with levels in other participants, CRP levels were higher among those living next to a busy road. Therefore, the study provides some support for a mechanistic link between proximity to traffic and deteriorating cardiovascular health. Future research should aim at identifying air pollution components responsible for the association between traffic intensity and systemic inflammation.

## Supplemental Material

(375 KB) PDF
