# Supplementary material for: Air Pollution from Road Traffic and Systemic Inflammation in Adults: A Cross-Sectional Analysis in the European ESCAPE Project
Source: Environ Health Perspect. 2015 Mar 27;123(8):785–91. doi: 10.1289/ehp.1408224 (PMC4529004; doi:10.1289/ehp.1408224)
Supplement: (375 KB) PDF [file ehp.1408224.s001.acco.pdf]

Environ Health Perspect
doi: 10.1289/ehp.1408224

Note to Readers: *EHP* strives to ensure that all journal content is accessible to all readers. However, some figures and Supplemental Material published in *EHP* articles may not conform to 508 standards due to the complexity of the information being presented. If you need assistance accessing journal content, please contact ehp508@niehs.nih.gov. Our staff will work with you to assess and meet your accessibility needs within 3 working days.

Supplemental Material

Air Pollution from Road Traffic and Systemic Inflammation in Adults: A Cross-Sectional Analysis in the European ESCAPE Project

Timo Lanki, Regina Hampel, Pekka Tiittanen, Silke Andrich, Rob Beelen, Bert Brunekreef, Julia Dratva, Ulf De Faire, Kateryna B. Fuks, Barbara Hoffmann, Medea Imboden, Pekka Jousilahti, Wolfgang Koenig, Amir A. Mahabadi, Nino Künzli, Nancy L. Pedersen, Johanna Penell, Göran Pershagen, Nicole M. Probst-Hensch, Emmanuel Schaffner, Christian Schindler, Dorothea Sugiri, Wim J.R. Swart, Ming-Yi Tsai, Anu W. Turunen, Gudrun Weinmayr, Kathrin Wolf, Tarja Yli-Tuomi, and Annette Peters

Table of Contents

Cohort descriptions

Biochemical measurements

Table S1. Associations of air pollution and indices of traffic intensity with C-reactive protein and fibrinogen in sensitivity analyses. Main models run adjusting for traffic noise levels, use of antihypertensive medication or statins, and excluding persons moving within 2 years before blood sampling. Effect estimates calculated for a change of 5 µg/m^3 in $PM_{2.5}$ and PM_{coarse}, 10 µg/m^3 in PM_{10} and NO_2, 20 µg/m^3 in NO_x, and 1x10^{-5}/m in $PM_{2.5}$ absorbance.

Figure S1. Cohort−specific and pooled exposure effects on CRP (main model). The size of the box indicating point estimate indicates the weight of the individual cohort on results; error bars represent 95% CIs.

Figure S2. Cohort−specific and pooled exposure effects on fibrinogen (main model). The size of the box indicating point estimate indicates the weight of the individual cohort on results; error bars represent 95% CIs.

Full acknowledgments

References

Cohort descriptions

The National FINRISK Study has been established in 1972; randomly selected representative population samples are recruited from different parts of Finland every five years (Vartiainen et al. 2010). For this study, three cross-sectional population surveys (1997, 2002, and 2007) and two study areas (cities of Helsinki/Vantaa and Turku/Loimaa region) were used. The KORA study is conducted within the framework of the Cooperative Health Research in the Region of Augsburg, and consists of four cross-sectional, population-representative surveys (Holle et al. 2005). ESCAPE includes two of the surveys, conducted in 1994-1995 and 1999-2001 in the city of Augsburg and two adjacent rural counties. The Heinz Nixdorf Recall study is an ongoing population-based, prospective cardiovascular cohort study that started in 2000, and includes 4,814 randomly selected participants from three large adjacent German cities (Essen, Mülheim/Ruhr, Bochum) (Schmermund et al. 2002). Health data from the baseline examination of the cohort (2000–2003) were used for the current analyses. For the SAPALDIA study, a random population sample across 8 geographic areas (Aarau, Basel, Davos, Geneva, Lugano, Montana, Payerne and Wald) was obtained in 1991 (Ackermann-Liebrich et al. 2005). For the current analyses, data of the 2002 follow-up (when biospecimens for CRP analyses were collected) from Basel, Lugano and Geneva are used. TwinGene is a substudy of SALT (Lichtenstein et al. 2006), which is based on a telephone interview 1998-2002, involving twins from the Swedish National Twin Registry (http://ki.se/en/research/the-swedish-twin-registry). In TwinGene, twins born before 1958 were contacted in the years 2004-2008 for the collection of biological samples. In the 60-year-olds cohort study (Wandell et al. 2007), a random sample of every third man and woman born between 1 July 1937 and 30 June 1938, and living in

Stockholm County, was invited to participation in a thorough health screening between August 1997 to March 1999. In total 4234 persons participated.

Biochemical measurements

In KORA, plasma CRP concentrations were measured with a high-sensitivity immunoradiometric assay from the blood samples taken during the 1994-1995 baseline visits (Hutchinson et al. 2000). Dade Behring N Latex High Sensitivity CRP mono assay on a Behring Nephelometer II (BN II) analyser was used for the blood samples of the later baseline visits (2000–2001). Fibrinogen plasma concentrations were determined by immunonephelometry on a BN II analyzer (Siemens, Marburg, Germany).

In HNR, high-sensitive CRP was measured with an automated nephelometer (BN II, Dade-Behring Inc, Deerfield, USA). Fibrinogen in plasma was measured with a Clauss method using an automated BCS-Analyzer (Dade-Behring Inc, Deerfield, USA). All analyses were performed in the central laboratory of the University Hospital of Essen.

In SAPALDIA, high-sensitive CRP serum levels were measured using Hitachi Modular Autoanalyser (Rotkreuz, Switzerland) and a latex-enhanced immunoturbidimetric assay (Roche Diagnostics, Mannheim, Germany).

In FINRISK, high-sensitive CRP was analyzed from the blood samples of the baseline visits of 1997 and 2007 using a latex immunoassay (Sentinel Diagnostics, Milan, Italy) with Architect c8000 analyser (Abbot Laboratories, Abbott Park, Illinois, US). Concerning the 2002 baseline visits, equipment by Orion Diagnostica (Espoo, Finland) was used for the immunoturbidometric assay coupled with the Optima analyser (Thermo Elektron, Waltham, MA, US). Fibrinogen was measured with the Clauss method using IL Test Fibrinogen-C kit and ACL300R equipment (Instrumentation Laboratory, Milan, Italy).

In TwinGene, high-sensitive CRP was determined using Synchron LX systems (Beckman Coulter, Brea, California, US). Fibrinogen was measured in the cohort of 60-year-olds by a functional spectrophotometric test (Boehringer Mannheim, Germany).

Table S1. Associations of air pollution and indices of traffic intensity with C-reactive protein and fibrinogen in sensitivity analyses. Main models run adjusting for traffic noise levels, use of antihypertensive medication or statins, and excluding persons moving within 2 years before blood sampling. Effect estimates calculated for a change of 5 μg/m^3 in $PM_{2.5}$ and PM_{coarse}, 10 μg/m^3 in PM_{10} and NO_2, 20 μg/m^3 in NO_x, and 1x10^{-5}/m in $PM_{2.5}$ absorbance.

	Traffic noise		Moving history		Antihypertensives		Statins	
**Exposures**	**% Diff[a] (95% CI)**	**P_{heter}[b]**	**% Diff[a] (95% CI)**	**P_{heter}[b]**	**% Diff[a] (95% CI)**	**P_{heter}[b]**	**% Diff[a] (95% CI)**	**P_{heter}[b]**
**CRP**								
$PM_{2.5}$	0.1 (-12.3, 14.3)	0.02	4.1 (-3.1, 11.8)	0.43	2.5 (-6.3, 12.0)	0.13	2.5 (-6.7, 12.7)	0.10
PM_{10}	0.3 (-5.4, 6.2)	0.74	1.3 (-4.2, 7.1)	0.88	0.7 (-4.3, 6.0)	0.92	0.9 (-4.2, 6.2)	0.92
PM_{coarse}	3.3 (-1.3, 8.1)	0.40	3.3 (-0.8, 7.6)	0.65	2.2 (-1.5, 5.9)	0.66	2.1 (-1.5, 5.9)	0.66
$PM_{2.5}$ absorbance	-0.9 (-7.2, 5.8)	0.50	1.7 (-4.7, 8.7)	0.85	0.2 (-5.5, 6.2)	0.85	0.7 (-5.1, 6.8)	0.81
NO_2	1.8 (-1.5, 5.3)	0.62	1.3 (-2.0, 4.7)	0.48	1.8 (-1.1, 4.9)	0.83	1.7 (-1.3, 4.7)	0.83
NO_x	3.4* (0.2, 6.7)	0.98	2.3 (-1.0, 5.8)	0.91	2.9* (0.0, 5.9)	0.99	2.5** (-0.4, 5.4)	0.99
**Traffic intensity at the nearest road (vehicles/day)**								
<1,000	Ref.		Ref.		Ref.		Ref.	
1,000-5,000	-1.5 (-6.4, 3.7)	0.80	-2.5 (-7.7, 2.9)	0.59	-1.7 (-6.3, 3.2)	0.62	-2.1 (-6.7, 2.7)	0.81
5,000-10,000	8.2* (1.0, 15.9)	0.99	5.1 (-2.3, 13.2)	0.54	2.8 (-6.9, 13.6)	0.28	4.5 (-2.0, 11.6)	0.58
>10,000	11.9* (3.4, 21.2)	0.75	9.9* (1.1, 19.5)	0.64	10.3* (2.4, 18.7)	0.98	9.9* (2.2, 18.3)	0.84
**Traffic load within 100 m on major roads (vehicles/day*m)**								
<500,000	Ref.		Ref.		Ref.		Ref.	
500,000-1,500,000	1.7 (-6.1, 10.2)	0.21	3.5 (-1.5, 8.8)	0.88	1.0 (-3.4, 5.7)	0.56	1.1 (-3.4, 5.8)	0.54
1,500,000-3,000,000	2.8 (-2.5, 8.4)	0.74	1.7 (-3.8, 7.4)	0.68	1.6 (-3.2, 6.6)	0.91	1.8 (-3.0, 6.9)	0.68
>3,000,000	2.5 (-4.1, 9.5)	0.32	2.8 (-4.3, 10.5)	0.27	2.7 (-4.0, 9.9)	0.21	1.9 (-4.3, 8.5)	0.27
**Fibrinogen**								
$PM_{2.5}$	0.0 (-1.9, 1.9)	0.63	0.2 (-1.4, 1.9)	0.60	0.1 (-1.4, 1.7)	0.36	-0.0 (-1.6, 1.6)	0.91
PM_{10}	0.2 (-1.3, 1.6)	0.44	-0.1 (-1.7, 1.5)	0.11	-0.1 (-1.5, 1.4)	0.24	-0.1 (-1.6, 1.3)	0.25
PM_{coarse}	-0.3 (-1.5, 0.9)	0.64	-0.1 (-1.5, 1.2)	0.13	-0.3 (-1.2, 0.7)	0.36	-0.3 (-1.2, 0.5)	0.39
$PM_{2.5}$ absorbance	-0.3 (-2.0, 1.4)	0.65	-0.0 (-2.3, 2.3)	0.23	0.0 (-1.4, 1.5)	0.49	-0.0 (-1.5, 1.4)	0.52
NO_2	0.3 (-1.4, 2.0)	0.10	0.3 (-1.1, 1.7)	0.04	0.4 (-0.7, 1.5)	0.11	0.3 (-0.8, 1.4)	0.12
NO_x	0.5 (-0.8, 1.8)	0.18	0.4 (-0.6, 1.5)	0.16	0.5 (-0.5, 1.4)	0.19	0.4 (-0.5, 1.3)	0.24

	Traffic noise		Moving history		Antihypertensives		Statins	
**Exposures**	**% Diff[a] (95% CI)**	**P_{heter}[b]**	**% Diff[a] (95% CI)**	**P_{heter}[b]**	**% Diff[a] (95% CI)**	**P_{heter}[b]**	**% Diff[a] (95% CI)**	**P_{heter}[b]**
**Traffic intensity at the nearest road (vehicles/day)**								
<1,000	Ref.		Ref.		Ref.		Ref.	
1,000-5,000	0.5 (-1.7, 2.6)	0.21	0.2 (-1.5, 1.8)	0.29	0.0 (-1.2, 1.2)	0.46	0.1 (-1.1, 1.3)	0.47
5,000-10,000	1.6** (-0.3, 3.5)	0.92	1.9* (0.1, 3.6)	0.98	1.6** (-0.1, 3.4)	0.89	1.7 (0.0, 3.5)	0.96
>10,000	0.9 (-1.2, 3.0)	0.61	0.8 (-1.1, 2.8)	0.74	0.6 (-1.2, 2.5)	0.73	0.6 (-1.3, 2.5)	0.72
**Traffic load within 100 m on major roads (vehicles/day*m)**								
<500,000	Ref.		Ref.		Ref.		Ref.	
500,000-1,500,000	0.2 (-1.0, 1.3)	0.42	0.3 (-0.8, 1.4)	0.52	0.0 (-1.1, 1.1)	0.48	0.1 (-1.0, 1.3)	0.33
1,500,000-3,000,000	0.9 (-0.4, 2.3)	0.63	1.0 (-0.3, 2.3)	0.95	0.7 (-0.5, 1.9)	0.89	0.8 (-0.5, 2.0)	0.88
>3,000,000	1.0 (-0.9, 2.9)	0.33	1.2 (-0.3, 2.7)	0.64	1.0 (-0.4, 2.4)	0.68	1.0 (-0.4, 2.4)	0.62

Main models run adjusting for traffic noise levels, use of antihypertensive medication or statins, and excluding persons moving within 2 years before blood sampling. Effect estimates calculated for a change of 5 μg/m^3 in $PM_{2.5}$ and PM_{coarse}, 10 μg/m^3 in PM_{10} and NO_2, 20 μg/m^3 in NO_x, and 1x10^{-5}/m in $PM_{2.5}$ absorbance.

[a]Percent difference. [b]p-value for heterogeneity.

*Effect estimates with p-values <0.05. **Effect estimates with p-values <0.1.

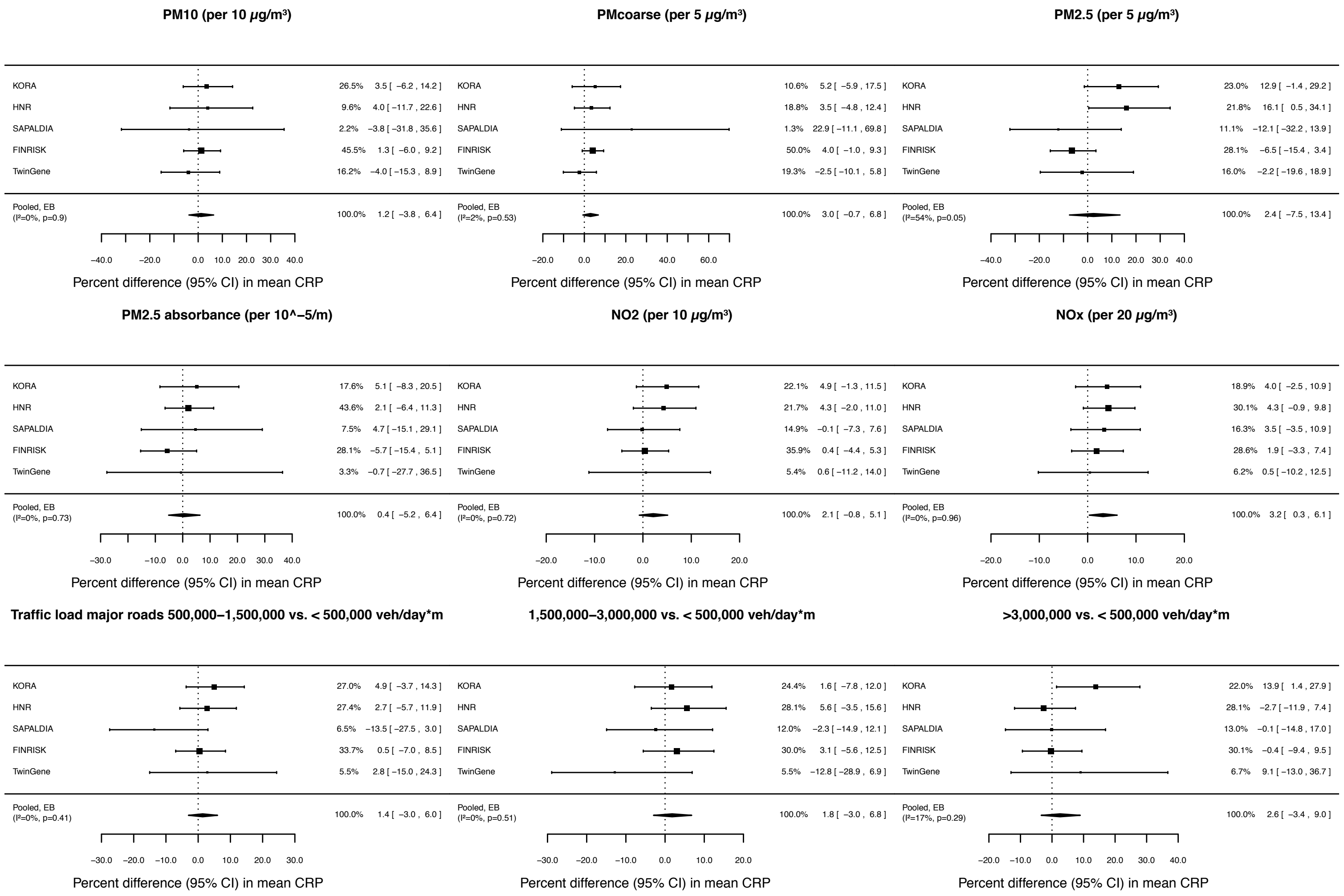

Figure S1. Cohort−specific and pooled exposure effects on CRP (main model). The size of the box indicating point estimate indicates the weight of the individual cohort on results; error bars represent 95% CIs.

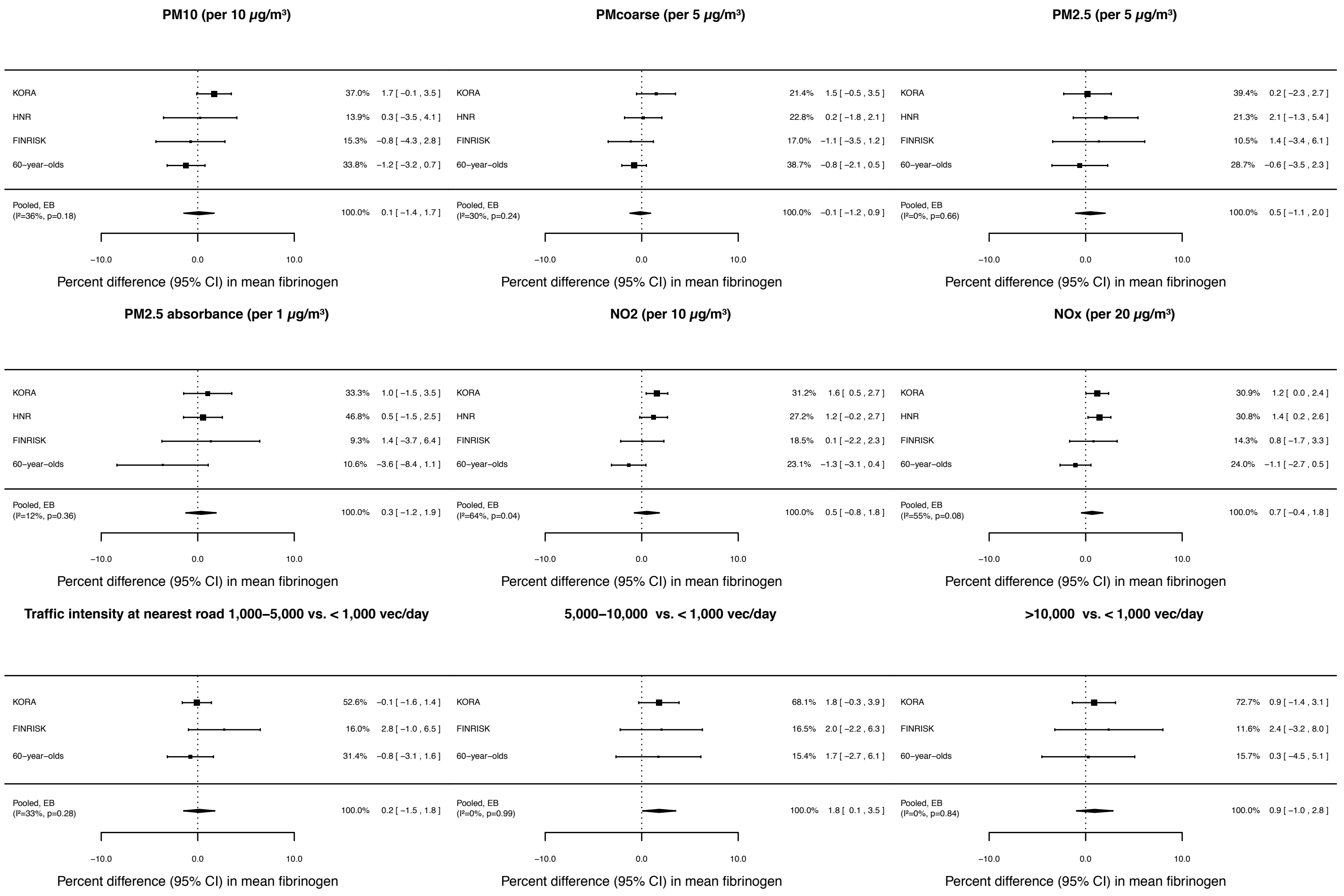

Figure S2. Cohort−specific and pooled exposure effects on fibrinogen (main model). The size of the box indicating point estimate indicates the weight of the individual cohort on results; error bars represent 95% CIs.

Full acknowledgments

HNR

We thank all study participants and the dedicated personnel of the Heinz Nixdorf Recall Study. We gratefully acknowledge the collaboration with K.-H. Jöckel, D. Grönemeyer, R. Seibel, K. Mann, L. Vollbracht, K. Lauterbach. We thank Anke Hüls for conducting the statistical analyses. We thank the North Rhine-Westphalia State Agency for Nature, Environment and Consumer Protection for providing road maps with traffic data and emission data from the reference sites for back-extrapolation. The study was supported by the Heinz Nixdorf Foundation [chairman: M. Nixdorf; former chairman: G. Schmidt (deceased)], the German Ministry of Education and Science, the German Research Foundation (DFG; projects JO-170/8-1, HO 3314/2-1, SI 236/8-1, and SI236/9-1).

SAPALDIA

The study was supported by the Swiss National Science Foundation (grants no 8 33CSCO-134276/1, 33CSCO-108796, 3247BO-104283, 3247BO-104288, 3247BO-104284, 9 3247-065896, 3100-059302, 3200-052720, 3200-042532, 4026-028099), the Federal Office for the Environment (FOEN), the Federal Office of Public Health, the Federal Office of Roads and Transport, the canton's government of Aargau, Basel-Stadt, Basel-Land, Geneva, Luzern, Ticino, Valais, and Zürich, the Swiss Lung League, the canton's Lung League of Basel Stadt/ Basel Landschaft, Geneva, Ticino, Valais and Zurich, SUVA, Freiwillige Akademische Gesellschaft, UBS Wealth Foundation, Talecris Biotherapeutics GmbH, Abbott Diagnostics, European Commission 018996 (GABRIEL), Wellcome Trust WT 084703MA. The study could not have

been done without the help of the study participants, technical and administrative support and the medical teams and field workers at the local study sites.

References

Ackermann-Liebrich U, Kuna-Dibbert B, Probst-Hensch NM, Schindler C, Felber Dietrich D, Stutz EZ, et al. 2005. Follow-up of the swiss cohort study on air pollution and lung diseases in adults (sapaldia 2) 1991-2003: Methods and characterization of participants. Soz Praventivmed 50:245-263.

Holle R, Happich M, Lowel H, Wichmann HE. 2005. Kora--a research platform for population based health research. Gesundheitswesen 67 Suppl 1:S19-25.

Hutchinson WL, Koenig W, Frohlich M, Sund M, Lowe GD, Pepys MB. 2000. Immunoradiometric assay of circulating c-reactive protein: Age-related values in the adult general population. Clin Chem 46:934-938.

Lichtenstein P, Sullivan PF, Cnattingius S, Gatz M, Johansson S, Carlstrom E, et al. 2006. The swedish twin registry in the third millennium: An update. Twin research and human genetics : the official journal of the International Society for Twin Studies 9:875-882.

Schmermund A, Mohlenkamp S, Stang A, Gronemeyer D, Seibel R, Hirche H, et al. 2002. Assessment of clinically silent atherosclerotic disease and established and novel risk factors for predicting myocardial infarction and cardiac death in healthy middle-aged subjects: Rationale and design of the heinz nixdorf recall study. Risk factors, evaluation of coronary calcium and lifestyle. Am Heart J 144:212-218.

Wandell PE, Wajngot A, de Faire U, Hellenius ML. 2007. Increased prevalence of diabetes among immigrants from non-european countries in 60-year-old men and women in sweden. Diabetes Metab 33:30-36.

Vartiainen E, Laatikainen T, Peltonen M, Juolevi A, Mannisto S, Sundvall J, et al. 2010. Thirty-five-year trends in cardiovascular risk factors in finland. Int J Epidemiol 39:504-518.
